# Using Transcranial Direct Current Stimulation to Augment the Effect of Motor Imagery-Assisted Brain-Computer Interface Training in Chronic Stroke Patients—Cortical Reorganization Considerations

**DOI:** 10.3389/fneur.2020.00948

**Published:** 2020-08-27

**Authors:** Effie Chew, Wei-Peng Teo, Ning Tang, Kai Keng Ang, Yee Sien Ng, Juan Helen Zhou, Irvin Teh, Kok Soon Phua, Ling Zhao, Cuntai Guan

**Affiliations:** ^1^Division of Neurology, Department of Medicine, National University Hospital, Singapore, Singapore; ^2^Yong Loo Lin School of Medicine, National University of Singapore, Singapore, Singapore; ^3^National Institute of Education, Nanyang Technological University, Singapore, Singapore; ^4^School of Exercise and Nutrition Sciences, Institute for Physical Activity and Nutrition, Deakin University, Melbourne, VIC, Australia; ^5^Institute for Infocomm Research (I^2^R), A*STAR, Singapore, Singapore; ^6^Department of Rehabilitation Medicine, Singapore General Hospital, Singapore, Singapore; ^7^Center for Sleep and Cognition, Center for Translational MR Research, Yong Loo Lin School of Medicine, Singapore, Singapore; ^8^Neuroscience and Behavioral Disorders Program, Duke-NUS Medical School, Singapore, Singapore; ^9^School of Medicine, University of Leeds, Leeds, United Kingdom; ^10^School of Computer Science and Engineering, Nanyang Technological University, Singapore, Singapore

**Keywords:** stroke, motor recovery, transcranial direct current stimulation, brain-computer interface, motor imagery

## Abstract

**Introduction:** Transcranial direct current stimulation (tDCS) has been shown to modulate cortical plasticity, enhance motor learning and post-stroke upper extremity motor recovery. It has also been demonstrated to facilitate activation of brain-computer interface (BCI) in stroke patients. We had previously demonstrated that BCI-assisted motor imagery (MI-BCI) can improve upper extremity impairment in chronic stroke participants. This study was carried out to investigate the effects of priming with tDCS prior to MI-BCI training in chronic stroke patients with moderate to severe upper extremity paresis and to investigate the cortical activity changes associated with training.

**Methods:** This is a double-blinded randomized clinical trial. Participants were randomized to receive 10 sessions of 20-min 1 mA tDCS or sham-tDCS before MI-BCI, with the anode applied to the ipsilesional, and the cathode to the contralesional primary motor cortex (M1). Upper extremity sub-scale of the Fugl-Meyer Assessment (UE-FM) and corticospinal excitability measured by transcranial magnetic stimulation (TMS) were assessed before, after and 4 weeks after intervention.

**Results:** Ten participants received real tDCS and nine received sham tDCS. UE-FM improved significantly in both groups after intervention. Of those with unrecordable motor evoked potential (MEP-) to the ipsilesional M1, significant improvement in UE-FM was found in the real-tDCS group, but not in the sham group. Resting motor threshold (RMT) of ipsilesional M1 decreased significantly after intervention in the real-tDCS group. Short intra-cortical inhibition (SICI) in the contralesional M1 was reduced significantly following intervention in the sham group. Correlation was found between baseline UE-FM score and changes in the contralesional SICI for all, as well as between changes in UE-FM and changes in contralesional RMT in the MEP- group.

**Conclusion:** MI-BCI improved the motor function of the stroke-affected arm in chronic stroke patients with moderate to severe impairment. tDCS did not confer overall additional benefit although there was a trend toward greater benefit. Cortical activity changes in the contralesional M1 associated with functional improvement suggests a possible role for the contralesional M1 in stroke recovery in more severely affected patients. This has important implications in designing neuromodulatory interventions for future studies and tailoring treatment.

**Clinical Trial Registration:** The study was registered at https://clinicaltrials.gov (NCT01897025).

## Introduction

Post-stroke recovery of upper extremity (UE) function remains a challenge. Less than 15% of stroke survivors with severe impairment experience complete motor recovery (1, 2). Intensive and repetitive practice is effective for motor recovery (3), but is labor-intensive and costly. More effective rehabilitation strategies that will deliver better functional outcomes without increasing cost of care are needed.

Motor imagery (MI), or mental practice is a mental rehearsal process of a specific movement without physical performance to enhance post-stroke upper extremity motor recovery (4–10). It has been demonstrated to be a safe, self-paced method to improve motor performance in athletes (6) and is effective in augmenting the effects of motor practice in stroke patients (7–9).

MI shares similar neural substrates with motor execution (11, 12). Functional neural changes induced by MI is similar to that of short-term motor learning (5) with corresponding changes in corticospinal excitability and reorganization of motor representation have been demonstrated with MI (4, 13).

Robot-assisted training is typically applied to deliver intensive, task-specific training in rehabilitation of motor function, but has also been used to provide appropriate sensorimotor integration through guidance of movement along a trajectory (14–18). The coupling of MI and robot-assisted arm movement through brain computer interface (MI-BCI) has been postulated to enhance sensorimotor integration by bridging the motor intent and providing appropriate somatosensory feedback through passive manipulation of the paretic arm, thereby guiding activity-dependent cortical plasticity through feedback on brain activity (19). Our previous studies of MI-BCI in chronic stroke demonstrated better improvement in motor function with fewer repetitions in the same time of training (20, 21). Others have found similar benefit using BCI-driven orthoses for rehabilitation of severe UE paresis (22).

Transcranial direct current stimulation (tDCS) is a non-invasive method of modulating corticospinal excitability by changing the firing threshold of neuronal membrane and modifying spontaneous activity according to the direction of current, such that cathodal tDCS decreases cortical excitability while anodal tDCS increases it (23–25). Good functional recovery has frequently been associated with a rebalancing of interhemispheric inhibition (17, 26). Based on this, cathodal tDCS is applied to the contralesional primary motor cortex (M1) and anodal tDCS to the ipsilesional M1 to enhance corticospinal excitability. This is the paradigm most frequently studied to enhance motor recovery after stroke (27–31), and has thus far yielded mixed results (32).

Additionally, tDCS has also been explored as a priming tool to improve the accuracy of BCI, both in healthy subjects (33, 34) and in stroke patients with mixed results (35, 36). We had previously reported the preliminary results of the first ever study to investigate the effect of a course of training with BCI-assisted motor imagery (MI-BCI) with tDCS priming (simultaneous anodal stimulation to the ipsilesional M1 and cathodal stimulation to the contralesional M1) prior to each session, compared to MI-BCI with sham tDCS, on recovery of chronic stroke patients with moderate to severe impairment (37). This population was chosen as they have the most difficulty engaging in active motor task training. The stimulation protocol was selected based on the intent to rebalance transcallosal inhibition, as suggested by previous studies (28, 30, 38). Clinical improvement was observed post-training, with online BCI accuracies being significantly better in the tDCS group, compared to the sham group.

The neurobiological principles that govern post-stroke recovery of motor function are incompletely understood. While task-specific training, and MI as an extension, is applied based on principles of activity-dependent cortical plasticity, and non-invasive brain stimulation is applied based on rebalancing of interhemispheric inhibitions, a more detailed understanding of the cortical reorganization associated with the combination of therapeutic modalities, and indeed of the recovery process itself, is required in order to tailor therapeutic approaches. TMS may be used to probe these changes in cortical excitability. Here we report the changes in cortical activity associated with this training protocol, which will inform the design of future studies.

## Participants and Methods

### Participants

A total of 42 participants were screened for eligibility for the study. All screening and study procedures were performed at the National University Hospital, Singapore. All partcipants provided voluntary, written informed consent in accordance with the Declaration of Helsinki. The study was approved by the National Healthcare Group Domain-Specific Review Board and was registered at https://clinicaltrials.gov (NCT01897025).

Patients 21–80 years old with a history of unilateral, single, hemorrhagic, or ischemic supratentorial stroke more than 9 months prior to enrolment, with upper extremity component of the Fugl-Meyer Assessment (UE-FM) (39) between 11 and 45 (moderate to severe motor impairment of arm) were eligible for inclusion. Participants were excluded based on the following: (1) inability to operate the MI-BCI system; (2) contraindications to TMS/tDCS including previous cranial surgeries, ferromagnetic implants, and seizures; (3) other factors affecting UE movement: severe pain in the affected UE that may be exacerbated by the use of the robotic device, major depression; (4) other neurological disorders.

### Sample Size Calculation

Sample size calculation was based on the minimal clinically important difference (MCID) score of the UE-FMA score, which is estimated to be 10 in a population of stroke patients with severe UE paresis (standard deviation of 10.73) (40). Based on a two-sided level of significance of 5% and a statistical power of 80%, the number of participants required is estimated to be 40 for a two-armed parallel-design study.

### Study Design and Randomization

This was a prospective, double-blinded, randomized controlled trial. Participants were randomized into real- or sham-tDCS intervention groups using a computer-generated stratified randomization approach. The randomization number generated was kept in a sealed envelope and was issued to the study coordinator before the start of intervention for each participant. Both the participants and assessors were blinded to the intervention that participants received.

### Intervention

Participants were initially screened for eligibility and ability to effectively activate the BCI system. Those who passed screening were randomly allocated to either real-tDCS or sham-tDCS group. Each received 10 sessions of real- or sham-tDCS, followed immediately by MI-BCI assisted robotic arm training. The intervention was conducted daily over 2 consecutive weeks.

### Transcranial Direct Current Stimulation (tDCS)

Direct current was delivered by a stimulator (NeuroConn, Germany) through rubber electrodes embedded in saline-soaked 50 ×70 mm^2^ sponge bags at an intensity of 1 mA. The anodal electrode was placed over the ipsilesional M1 and the cathodal electrode was placed over the contralesional M1. Stimulation intensity was ramped up to 1 mA over 30 s and maintained for 20 min, before ramping down. Sham-tDCS was delivered by similarly ramping up to 1 mA but maintained for only 20 s to give participants the same scalp sensation, before ramping down (29). tDCS intervention lasted for 20 min for both groups so that participants were blinded to their group allocation.

### Motor Imagery—Assisted Brain-Computer Interface (MI-BCI) Coupled With Robotic Arm Training

The MI-BCI protocol has been detailed in previous publication (37). In short, 27-channel electroencephalogram (EEG) signals were recorded by NuAmp EEG amplifier (Compumedic, Germany). The Inmotion^2^ MIT-Manus robot (Interactive Motion Technologies, MA, USA) was used to provide unrestricted unilateral passive and active shoulder and elbow movements in the horizontal plane (41). Visual feedback from the screen indicated the success or failure of MI detection for each MI task. Once motor intention was successfully detected, the robot-assisted motion would be triggered according to the clock exercise therapy of the MIT-Manus robot (42).

### Outcome Measures

All outcome measures were performed within 1 week prior to commencement of the intervention (PRE), within 1 week after the intervention was completed (POST1), and again at 4 weeks post-intervention (POST2).

### Upper Extremity Motor Function Assessment

The UE-FM (39) was the primary outcome measure in this study. UE-FM assessment was performed by a single senior occupational therapist who was blinded to the group allocation.

### Corticospinal Excitability Measurement by TMS

TMS measurement of corticospinal excitability was performed by a single research assistant. Resting motor threshold (RMT), short intra-cortical inhibition (SICI) and short intra-cortical facilitation (SICF) were measured using transcranial magnetic stimulation of the primary motor cortex, with participant sitting upright in a chair with back supported, looking forward, with both forearms resting comfortably on pillows and elbows supported at 90°. Motor-evoked potentials (MEPs) were recorded from the abductor pollicus brevis (APB) via surface electrodes in a belly-tendon arrangement, by Medelec Synergy Electromyography (EMG) system (VIASYS Healthcare, UK). Single- and paired-pulse TMS were delivered through a 70 mm figure-of-eight coil using Bistim 200^2^ (Magstim Co., UK). The coil was placed on the scalp with the handle pointing posteriorly at a 45° between the coronal and sagittal planes. The optimal scalp position for activating APB was determined from initial exploration over a 1-cm grid marked on a swimming cap worn over the head.

RMT was defined by the lowest intensity eliciting peak-to-peak MEP amplitude of 50 μV, in at least five out of 10 trials from single-pulse TMS stimulation (43). SICI and SICF were measured using paired-pulse stimulation with a conditioning stimulus of 80% of RMT followed by a test stimulus of 120% of RMT. MEPs were recorded with different inter-stimulus intervals (ISIs). ISI of 2 ms typically induces SICI while ISIs of 10 and 15 ms reflect SICF (44, 45).

Adverse effects were monitored using a questionnaire documenting pain and discomfort at the stimulation site. The Beck Depression Inventory, the Fatigue Severity Scale, and the forward and backward digit span tests were administered for possible psychological and cognitive changes which may be potential confounders.

### Statistical Analysis

All statistical analysis was carried out using the IBM SPSS version 23 software. Linear mixed model with an unstructured covariance matrix and Bonferroni adjustment was used to compare differences between the two intervention groups (real-tDCS vs. sham tDCS) and among three time points (PRE, POST1, and POST2). Differences in the baseline UE-FM between two groups were analyzed by the student *t*-test. Chi-Square test was used to compare differences between categorical data. Correlation of non-parametric data was analyzed using Spearman Correlation. *P* <0.05 was set as the level for statistical significance.

We further analyzed the data from participants whose MEP of the ipsilesional M1 were unrecordable even at maximal TMS stimulator output (MEP-). Absence of MEP is associated with poorer functional outcomes in stroke patients (46).

## Results

**Figure 1:**
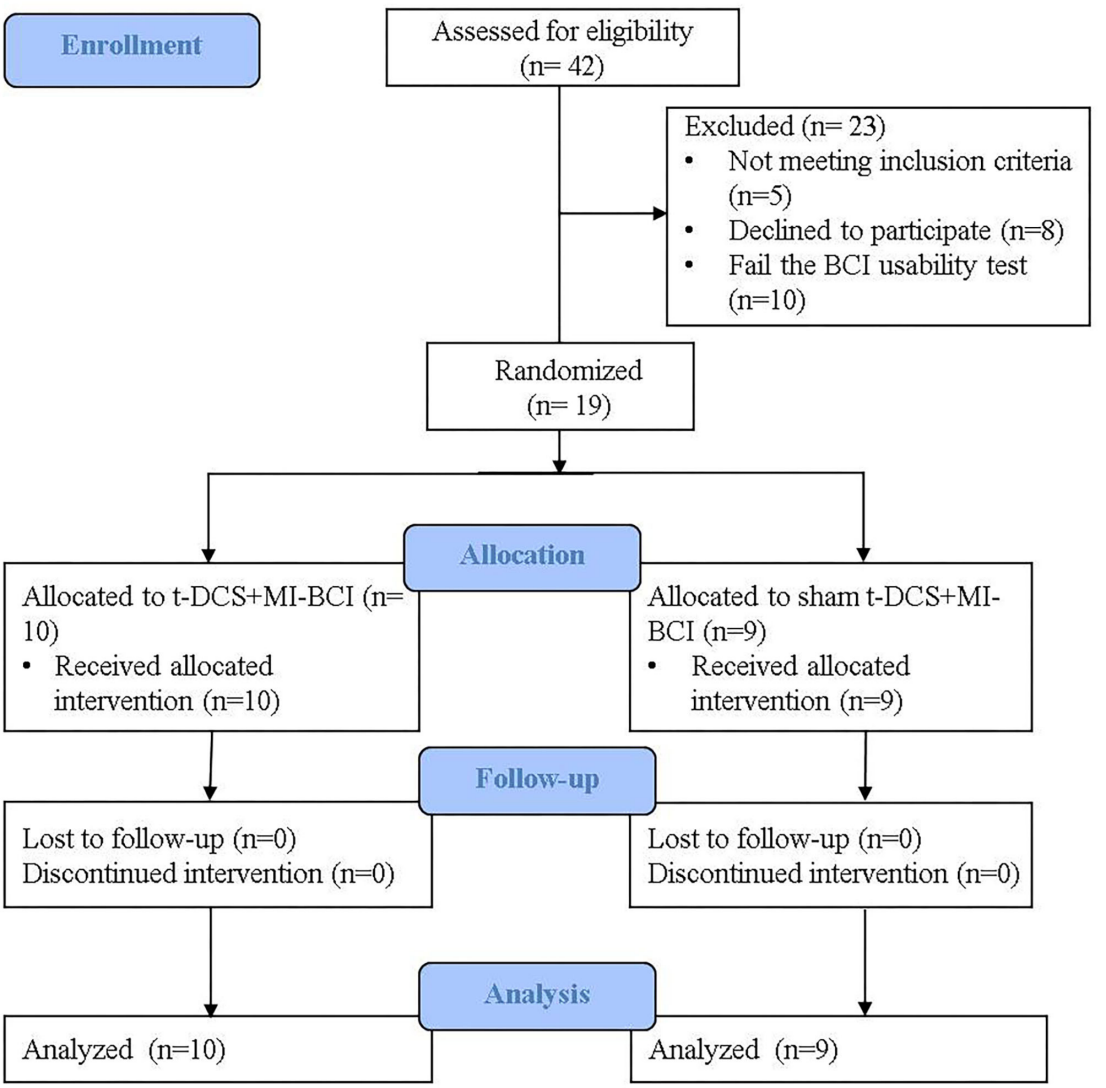
CONSORT flow diagram. Forty-two participants were screened. Nineteen participants completed the intervention and follow-up evaluation and were included in the final analysis−10 in the real-tDCS group, nine in the sham-tDCS group.

We were not be able to reach the planned recruitment target. Of 42 chronic stroke patients who underwent screening, 19 were recruited, 10 to the real-tDCS group and nine to the sham-tDCS group. All completed the intervention and the follow-up evaluations (Figure 1). Six patients in the real-tDCS group and five patients in the sham-tDCS group were MEP- at the baseline. There was no statistical difference between groups in baseline demographic and stroke characteristics (Table 1), as has been reported previously (37).

**Table 1:** Clinical-demographics characteristics.

**Group**	**Gender**	**Age (year)**	**Stroke duration (months)**	**Affected hemisphere**	**Affected region**	**Ischaemic/Haemorrhagic**	**Comorbidities**	**UE-FM**	**MEP**
Real tDCS	M	29	11	R	Parietal	I	hypertension, hyperlipidaemia	51	+
	M	54	28	L	CR, IC	I	hypertension, hyperlipidaemia, DM	29	+
	F	38	29	R	BG	H	DM, Turner's syndrome	38	-
	F	60	51	R	BG H. extending to temporal and CR	H	hypertension	26	+
	F	48	49	L	BG H. extending to frontal and CR	H	hypertension	39	-
	M	59	13	L	MCA territory subcortical	I	DM, hypertension, hyperlipidaemia, IHD	31	+
	M	65	27	L	CR	I	DM, hypertension, hyperlipidaemia	41	-
	F	57	10	L	BG, CR	H	none	40	-
	M	47	9	R	MCA territory subcortical	I	Atrial fibrillation	30	-
	M	65	86	R	CR, IC, BG	I	DM, hypertension, hyperlipidaemia	28	-
Mean ± SD	6M/4F	52.2 ± 11.8	31.3 ± 24.5	5L/5R	-	6I/4H	-	35.3 ± 7.8	4+/6-
Sham tDCS	M	51	44	R	MCA territory subcortical	I	IHD, hyperlipidaemia,	33	+
	M	39	25	L	Subcortical (intracranial large vessel disease)	I	Acute myeloid leukemia	36	-
	M	59	52	R	BG	H	Hypertension hyperlipidaemia	41	+
	F	70	19	R	MCA territory subcortical	I	Hyperlipidaemia, rheumatic heart disease	23	-
	M	59	44	R	MCA territory subcortical	I	DM, hypertension, hyperlipidaemia	29	-
	M	58	29	L	MCA territory subcortical	I	Hypertension, hyperlipidaemia	28	-
	M	58	25	R	BG	H	Hypertension, hyperlipidaemia	20	+
	M	47	10	L	Thalamus	I	Hypertension, hyperlipidaemia	40	-
	M	67	52	R	CR	I	-	43	+
Mean ± SD	8M/1F	56.4 ± 9.6	33.3 ± 15.1	3L/6R	-	7I/ 2H	-	32.6 ± 8.1	4+/5-
Statistics	χ_(1)_ = 2.04, *p* = 0.15	*t* = 0.86, *p* = 0.40	*t* = 0.21, *p* = 0.83	χ_(1)_ = 0.69, *p* = 0.40	-	χ_(1)_ = 0.54, *p* = 0.46	-	*t* = 0.75, *p* = 0.46	χ_(1)_ = 0.84, *p* = 1.00

*No statistical differences in demographic data were found between the real-tDCS and the sham-tDCS group, including the initial UE function. Data was analyzed by the independent student t-test or Pearson's Chi-Square test. Data shows Mean ± SD or number of cases*.

*M, male; F, female; L, left; R, right; CR, corona radiata; IC, internal capsule; BG, basal ganglia; MCA, middle cerebral artery; I, ischemic stroke; H, haemorrhagic stroke; DM, Diabetes Mellitus; IHD, Ischemic Heart Disease; UE-FM, Upper extremity sub-scale of the Fugl-Meyer Assessment; +, MEP is recordable from the ipsilesional M1; -, MEP is not recordable from the ipsilesional M1*.

### Upper Extremity Motor Function Measurement

**Figure 2:**
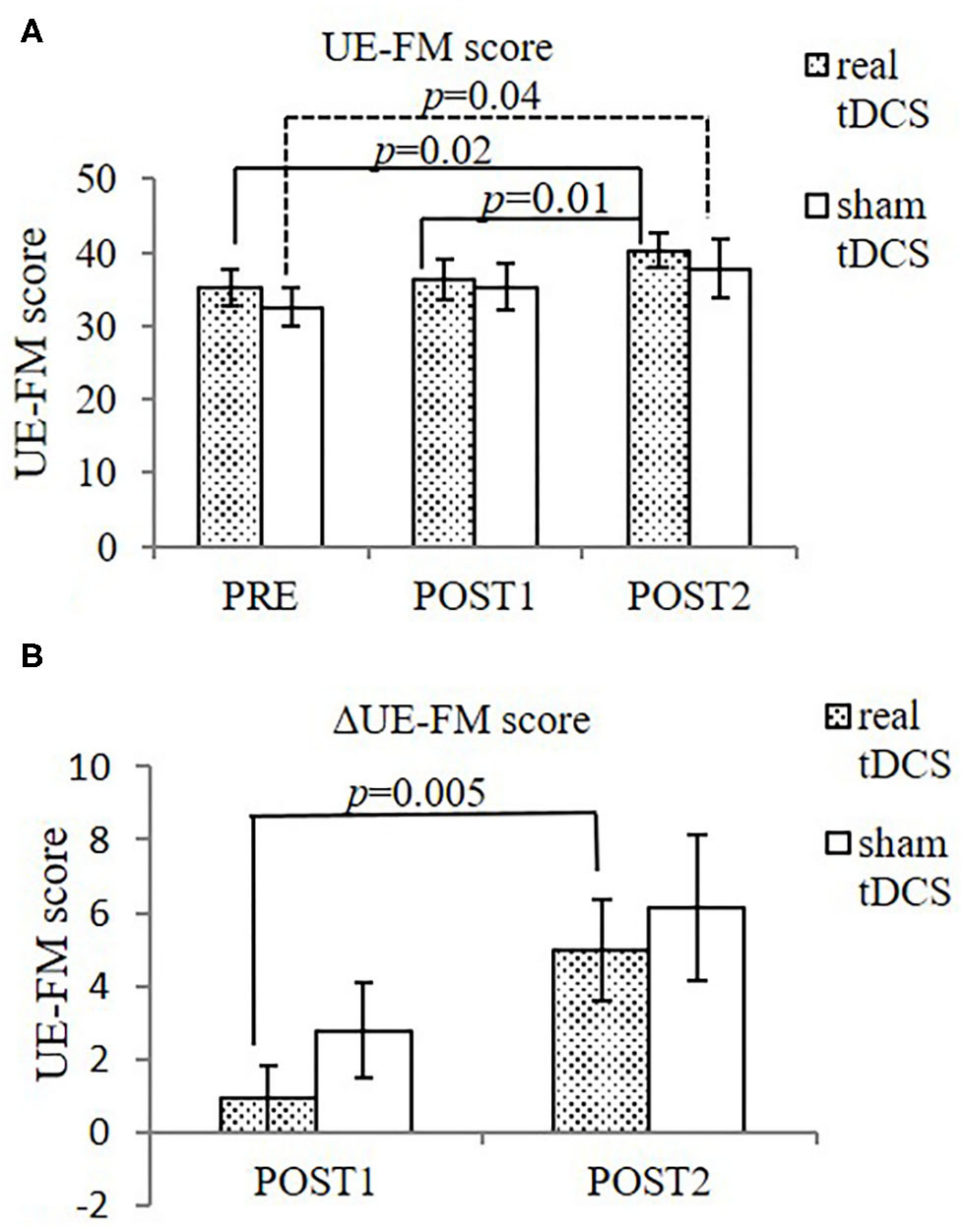
UE-FM score **(A)** and ΔUE-FM **(B)** in both groups. **(A)** Both groups improved significantly in UE-FM at POST2 after intervention (*n* = 10 for real-tDCS group, *n* = 9 for sham-tDCS group). Between group difference was not statistically significant. **(B)** ΔUE-FM (changes in UE-FM score compared to PRE) was significantly higher at POST2, compared to POST1 in the real-tDCS group, not in the sham group. Data shows mean ± SEM.

Both the real- and sham-tDCS groups improved significantly in the UE-FM after intervention [from 35.3 ± 7.8 (PRE) to 36.2 ± 8.8 (POST1) and 40.3 ± 7.8 (POST2), *F* = 7.64; *p* = 0.01 for real-tDCS group; from 32.6 ± 8.1 (PRE) to 35.3 ± 9.6 (POST1) and 37.8 ± 11.4 (POST2), *F* = 4.85; *p* = 0.04 for sham group], with no statistically significant difference between groups (*F* = 0.23, *p* = 0.64). The analysis on ΔUE-FM (UE-FM compared to pre-intervention) was previously reported (37). ΔUE-FM was significantly higher at POST2, compared to POST1 in real-tDCS group [from 0.9 ± 3.0 (POST1) to 5.0 ± 4.4 (POST2), *F* = 13.64; *p* = 0.005], but not in the sham group [from 2.8 ± 4.0 (POST1) to 6.1 ± 5.7 (POST2), *F* = 4.45; *p* = 0.07] (Figure 2).

**Figure 3:**
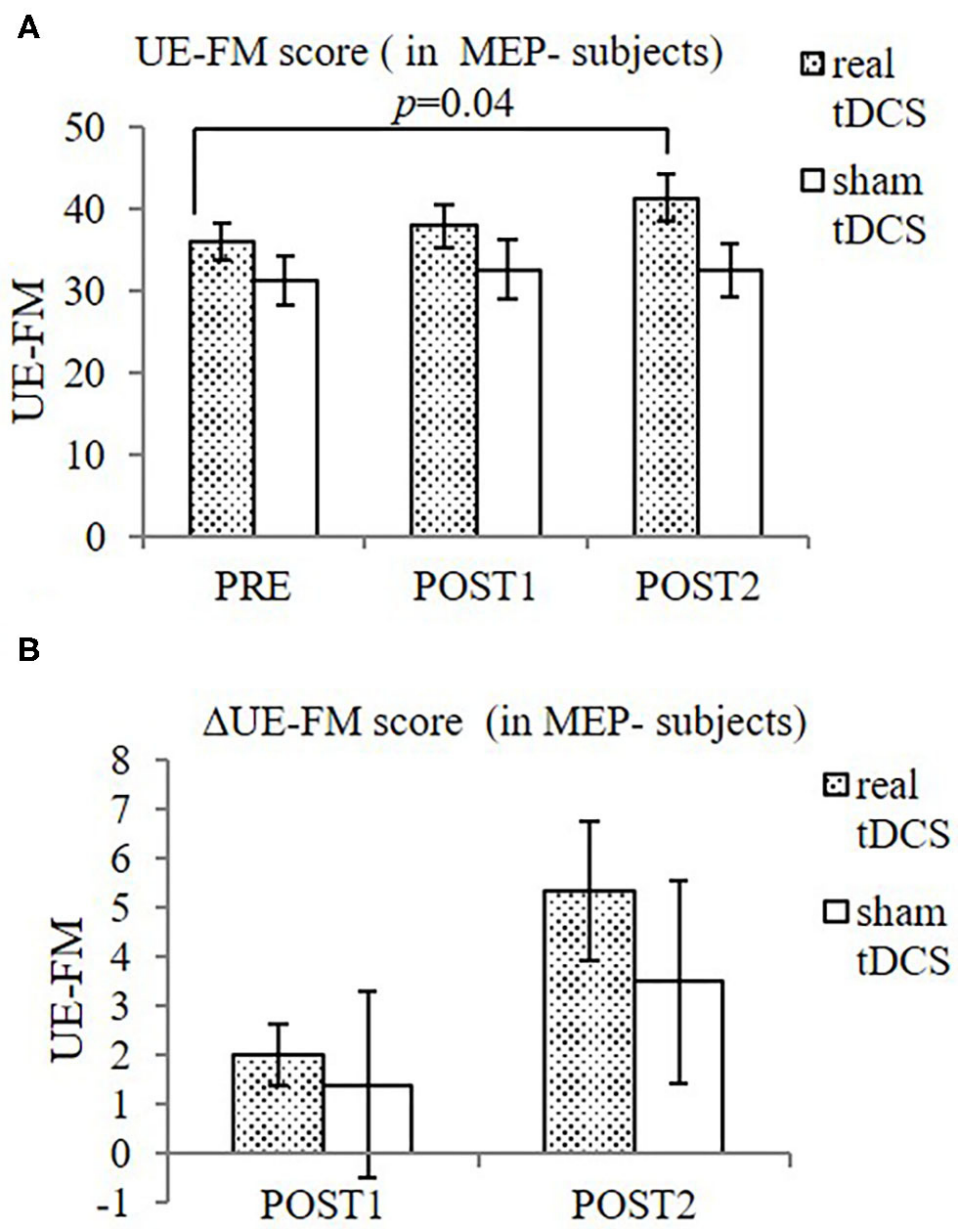
UE-FM **(A)** and ΔUE-FM **(B)** in both groups in MEP- participants. **(A)** Significant improvement in UE-FM in the real-tDCS group (*n* = 6), but not the sham-tDCS group (*n* = 5). **(B)** No significant difference in ΔUE-FM was shown between groups, or over time. Data shows mean ± SEM.

When MEP- participants were considered alone, significant improvement in UE-FM was found only in the real-tDCS group [from 36.0 ± 5.5 (PRE) to 38.0 ± 6.4 (POST1) and 41.3 ± 7.1 (POST2), *F* = 9.71, *p* = 0.02], but not in the sham-tDCS group [from 31.2 ± 6.8 (PRE) to 32.6 ± 8.3 (POST1) and 32.5 ± 6.5 (POST2), *F* = 0.88, *p* = 0.50] (Figure 3).

### Neurophysiological Outcome Measures—RMT

**Figure 4:**
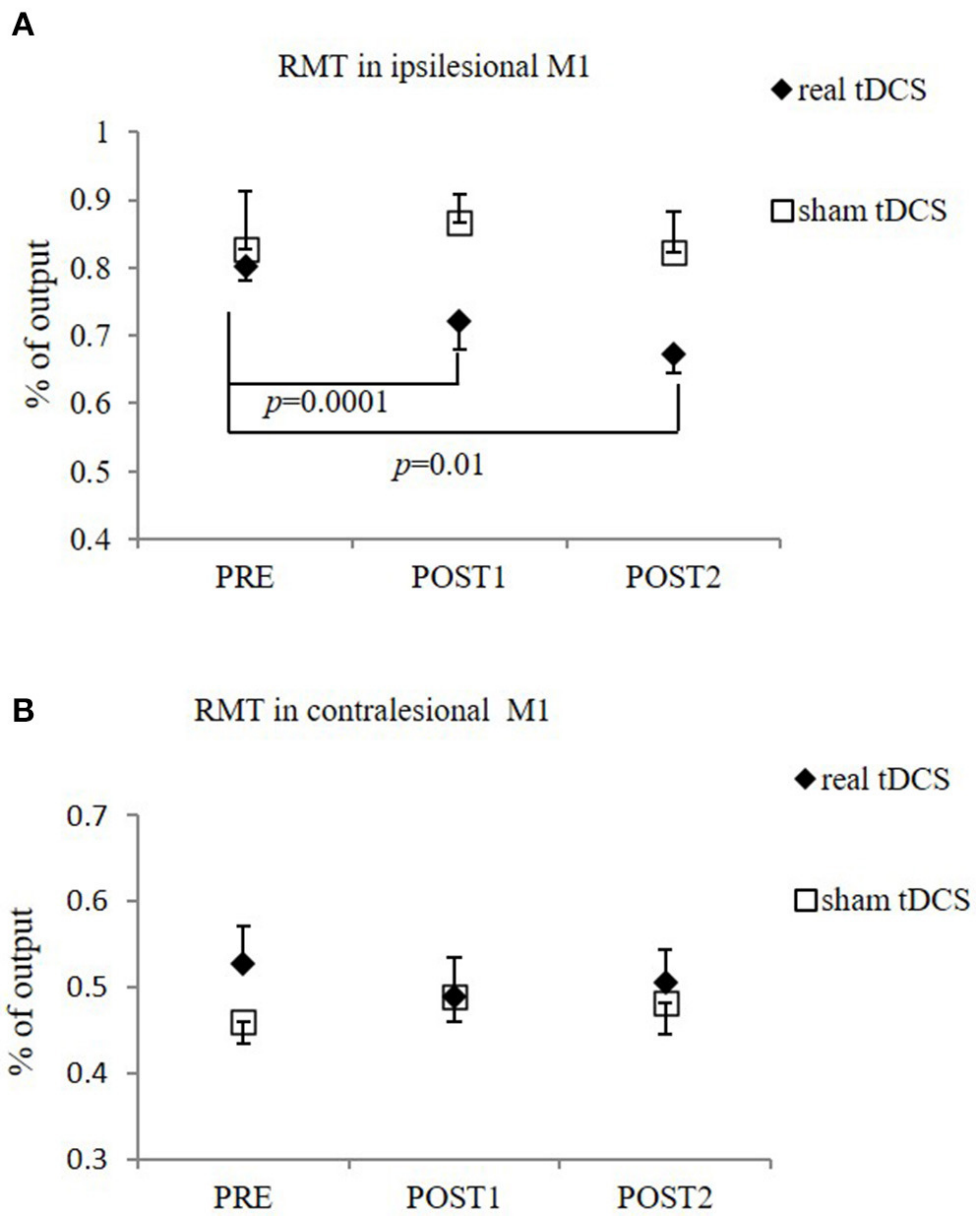
RMT in the ipsilesional M1 **(A)** and contralesional M1 **(B)**. **(A)** Significant reduction in RMT in ipsilesional M1 in real-tDCS (*n* = 6) group at POST1 and POST2, compared to PRE, but not in sham-tDCS group (*n* = 5). The overall difference between groups was statistically significant. **(B)** No significant difference in RMT in contralesional side between two groups (*n* = 10 for real-tDCS group, *n* = 9 for sham-tDCS group), or over time. Data shows mean ± SEM.

There was significant difference in RMT of the ipsilesional M1 over time in real-tDCS group [from 0.80 ± 0.04 (PRE) to 0.72 ± 0.07 (POST1) and 0.67 ± 0.06 (POST2), *F* = 12.67; *p* = 0.00], but not in the sham-tDCS group [from 0.83 ± 0.17 (PRE) to 0.87 ± 0.08 (POST1) and 0.82 ± 0.12 (POST2), *F* = 3.00; *p* = 0.19]. *Post-hoc* Bonferroni test showed that RMT of the real-tDCS group was significantly lower at POST1 and POST2, compared to PRE (*p* = 0.0001 and 0.01, respectively). The overall difference between real and sham groups was statistically significant (*F* = 15.12; *p* = 0.01). No significant within- and between-group differences in the RMT were found in the contralesional M1 (Figure 4).

### Neurophysiological Outcome Measures—SICI and SICF in the Contralesional M1

**Figure 5:**
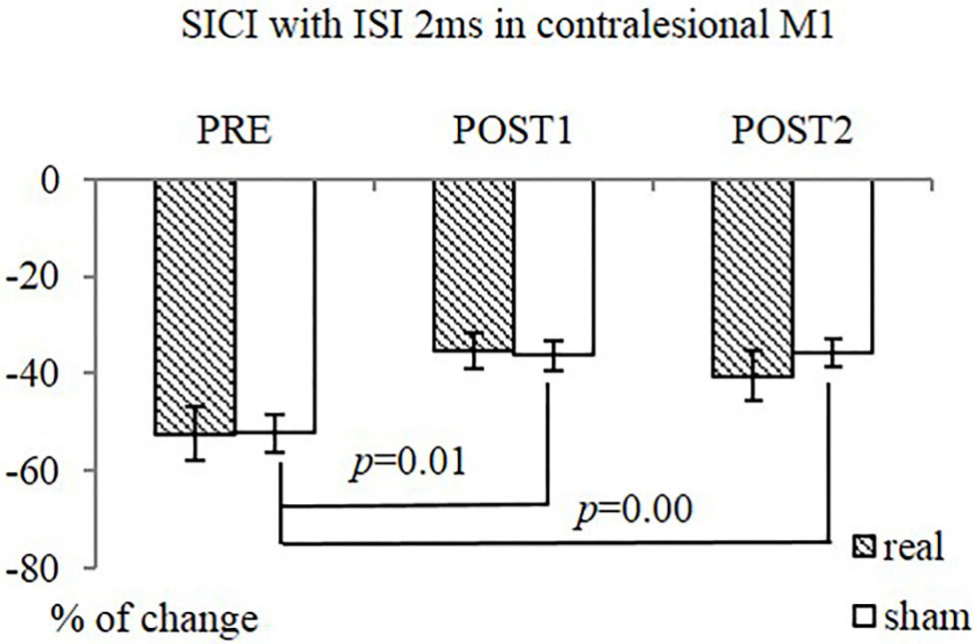
SICI (ISI 2ms) in contralesional M1 in both groups. The sham-tDCS group (*n* = 9) had significantly reduced SICI_2ms_ at POST1 and POST2, compared PRE. No difference over time was observed in real-tDCS group (*n* = 10). Between-group difference was not significant.

The interventions reduced SICI in the contralesional M1 significantly, as measured at ISI of 2 ms (SICI_2ms_), in the contralesional M1 (*F* = 9.34, *p* = 0.00), when both groups were combined. The sham-tDCS group had significantly reduced SICI_2ms_ following intervention [from −52.2 ± 11.6 (PRE) to −36.3 ± 8.3 (POST1) and −35.9 ± 8.7 (POST2), *F* = 27.15, *p* = 0.00]. The difference in the real-tDCS group was not significant, as was the difference between groups (Figure 5). There was no significant difference in SICF between groups, or over time.

### Relationship Between UE-FM and Contralesional Corticospinal Excitability

Spearman's correlation was used to investigate the relationships between clinical outcome measures (UE-FM) and contralesional corticospinal excitability. Correlation was found between the UE-FM score and the contralesional RMT such that a higher UE-FM score was associated with lower contralesional RMT (*r* = −0.315, *p* = 0.019). A lower UE-FM score was also associated with greater changes in contralesional SICI_2ms_ (ΔSICI_2ms_) (*r* = −0.420, *p* = 0.012) (Table 2). When MEP- participants were considered alone, a greater reduction in contralesional RMT (ΔRMT, difference from PRE) was associated with greater improvement in UE-FM score (ΔUE-FM, difference from PRE) (*r* = −0.463; *p* = 0.034) (Table 2).

**Table 2:** Correlation between UE-FM and corticospinal excitability in the contralesional M1.

	**All participants**			
	**UE-FM**		**ΔUE-FM**	
	**Correlation**	***p*-value**	**Correlation**	***p*-value**
	**coefficient**		**coefficient**	
Contralesional RMT	−0.315*	0.019	−0.174	0.309
ΔRMT	−0.325	0.053	−0.14	0.414
SICI_2ms_	0.127	0.36	0.022	0.902
ΔSICI_2ms_	−0.420*	0.012	−0.057	0.744
	**MEP** — **participants**			
Contralesional RMT	−0.204	0.263	−0.201	0.382
ΔRMT	−0.369	0.099	−0.463*	0.034
SICI_2ms_	0.180	0.332	−0.220	0.352
ΔSICI_2ms_	−0.415	0.069	−0.110	0.645

UE-FM score is negatively correlated with RMT and ΔSICI_2ms_ (changes in SICI_2ms_ compared to PRE). When MEP- participants were considered alone, moderate correlation was found between a decrease in RMT (ΔRMT, changes in RMT compared to PRE) in the contralesional M1, and an improvement in UE-FM score (ΔUE-FM). *p <0.05.

### Side Effects of Intervention

No complications of tDCS or MI-BCI were reported by participants during and after intervention. There was no within- and between-group difference in the forward and backward digit-span, the Beck Depression Inventory and the Fatigue Severity Scale.

## Discussion

In this preliminary study, both the real- and sham-tDCS groups improved significantly in UE function with MI-BCI training. The intervention of MI-BCI with tDCS prior to it was safe and well-tolerated by our patients. MI-BCI training was again demonstrated to improve motor function despite initial moderate to severe motor impairment, with gains continuing up to 4 weeks post-intervention, which were greater in extent in the real-tDCS group.

Previous evidence suggests that modulation of cortical excitability with tDCS prior to task training may result in greater improvements in motor outcomes (27, 29–31, 47). A recent systematic review suggested that response to contralesional inhibitory neuromodulation may be affected by timing—while smaller studies demonstrated a definite effect in UE stroke recovery in the post-acute stage, one large study in the chronic stage did not demonstrate improved UE function (48). Whether this was because of the heterogeneity of the participants or the decreased response to modulation in the chronic phase is debatable. The lack of clear, additional clinical benefit in adding tDCS to MI-BCI training in our study may be attributed to the small sample size which had not reach our planned recruitment target, and the relatively short training period in the context of chronic stroke. Indeed, there was a trend toward significant difference between the tDCS and sham groups, in favor of the tDCS group. With the inclusion of more patients and a longer training duration, we may see a significant effect between groups.

Patient selection and the stimulation protocol selected, may have also contributed to the lack of observable difference. Recent literature suggests that recovery of motor function post-stroke follows a relatively predictable “proportional recovery rule” (49–51), which describes the potential for recovery ~70% of the maximum possible. Integrity of the corticospinal tract is an important factor determining adherence to this rule (“fitters”) (49, 51). Those without intact corticospinal tracts tend to be “non-fitters” to the rule, have more severe impairments and show poorer recovery.

Our population of patients were mostly those with undetectable MEPs at the time of recruitment. It has been suggested that such “non-fitters” may adopt different neural mechanisms to achieve recovery compared to those with greater integrity of the corticospinal tracts. Di Pino et al. proposed a bimodal balance-recovery model in which those with high structural reserves would achieve best recovery through rebalancing of interhemispheric inhibition, while those with low structural reserves (i.e., Larger area of damage and more severe impairment) may achieve better outcomes through promotion of vicarious activity in the unaffected hemisphere (52). We based our choice of tDCS protocol on the intent to rebalance interhemispheric inhibition, based on previous literature, without considering the integrity of the corticospinal tract. This may have contributed to the lack of observed efficacy. Indeed, more recent studies have applied a stratified approach using clinical and functional imaging cut-offs to facilitate selection of a tailored stimulation protocol (facilitation vs. inhibition of the contralesional hemisphere) (53).

Notwithstanding, we were able to observe a clinical improvement in both groups. We found a correlation between the degree of impairment in the stroke-affected arm and the degree of change in intracortical inhibition on the non-lesioned hemisphere. Furthermore, in the MEP- group, improvement in function was associated with increased corticospinal excitability on the contralesional motor cortex. These findings suggest a role of the contralesional hemisphere in the recovery of motor function post-stroke.

Cortical reorganization, with an increase of excitability of the contralesional hemisphere has been observed repeatedly following stroke (54–56). But the significance of this in motor recovery remains uncertain (57). Inhibition of the contralesional hemisphere has been shown to lead to worsening of function in the stroke-affected limb in both animals and humans (58, 59). Whether bilateral activation during task performance reflects poorer outcomes is debated. Some have found that bilateral activation portends poor outcome (60), while others have found it persists in well-recovered stroke patients (61, 62). Of note, the extent of contralesional activation relates to the degree of motor skill challenge (63) and would be an important consideration in relation to the extent of motor impairment. In terms of exploring alternative approaches to non-invasive brain stimulation, the contralesional premotor cortex has been identified as a promising target in preliminary studies to augment recovery for the more severely affected stroke patients, while little effect has been demonstrated with facilitation or perturbation of the contralesional M1 (53, 59).

The mechanism by which the contralesional motor cortex may facilitates motor recovery is a matter of active investigation. Indeed, the interhemispheric inhibition in stroke recovery has been questioned in recent studies. Premovement interhemispheric inhibition in a group of mild to moderately impaired stroke patients was found to be preserved early post-stroke and only became abnormal in the chronic phase, with no cross-sectional correlation with functional recovery (64). Studies using TMS have demonstrated that the increased contralesional M1 excitability is not causally related to the decreased transcallosal inhibition from the ipsilesional M1 (56). But rather, a decrease in intracortical inhibition as measured by SICI, which reflects the activity of GABA_A_ergic interneurons (65), may mediate the contralesional M1 reorganization. The relatively suppressed inhibitory effect of the conditioning stimulus at higher intensity suggests a shift in the balance of excitatory and inhibitory activity toward an increase in contralesional excitatory activity (55). Such a reduction in SICI in the subacute period post-stroke is associated with significant functional improvement and may reflect the unmasking of latent networks critical for cortical reorganization (55, 66). Our finding that clinical improvement correlated with a reduction in contralesional SICI suggests that such a decrease in GABA_A_-mediated inhibition may also play a role in contralesional reorganization associated with functional improvement, even in the chronic phase of stroke. Further investigation is required to ascertain this.

Finally, with regard to how tDCS may augment MI-BCI training, we had previously demonstrated an increase in MI detection accuracy with real-tDCS compared to sham-tDCS (21). A higher accuracy for classifying MI was observed in stroke participants following bi-hemispheric tDCS (67). Others have demonstrated a modulation of event-related desynchrony during MI with tDCS, which may enhance BCI accuracy and contribute to more effective training (35, 68, 69).

Anodal tDCS may also exert influence on training efficacy through enhancing implicit motor learning (24), or by improving attention (70). The greater delayed improvement demonstrated by the tDCS group may also reflect NMDA-dependent long-term changes in synaptic efficacy, an important mechanism underlying learning, and memory processes (23, 71).

In conclusion, MI-BCI resulted in significant UE improvement in chronic stroke patients with moderate to severe impairment. A trend toward better outcomes in the real-tDCS group was observed with significant benefit seen in the MEP- group. Future studies with more participants should focus on elucidating the specific neural mechanisms underlying motor recovery and the interaction of individual and stroke factors, tailoring neuromodulatory interventions using a stratified approach, and determining the optimal approach to combining MI-BCI with non-invasive brain stimulation to enhance motor recovery.

## Data Availability Statement

All datasets generated for this study are included in the article/supplementary material.

## Ethics Statement

The studies involving human participants were reviewed and approved by National Healthcare Group Domain-Specific Review Board. The patients/participants provided their written informed consent to participate in this study.

## Author Contributions

EC, YN, and CG designed and directed the study. CG and KA developed the MI-BCI EEG analysis system. KP and LZ carried out the clinical trial procedures including MI-BCI training and trial coordination. EC, W-PT, and NT contributed to the data analysis and to the writing of the manuscript. All authors contributed to the article and approved the submitted version.

## Conflict of Interest

The authors declare that the research was conducted in the absence of any commercial or financial relationships that could be construed as a potential conflict of interest.

## Abbreviations

APB: abductor pollicus brevis
BCI: brain-computer interface
EEG: electroencephalogram
ISI: inter-stimulus interval
MEP: motor evoked potential
MI: motor imagery
MI-BCI: motor imagery-assisted brain-computer interface
POST1: within 1 week after the intervention
POST2: 4 weeks post-intervention
PRE: 1 week prior to commencement of the intervention
RMT: Resting motor threshold
SICF: intracortical facilitation
SICI: Short intra-cortical inhibition
tDCS: Transcranial direct current stimulation
TMS: transcranial magnetic stimulation
UE: upper extremity
UE-FM: upper extremity component of the Fugl-Meyer Assessment.
