# Corrigendum: Using Transcranial Direct Current Stimulation to Augment the Effect of Motor Imagery-Assisted Brain-Computer Interface Training in Chronic Stroke Patients—Cortical Reorganization Considerations

**DOI:** 10.3389/fneur.2020.605141

**Published:** 2020-11-06

**Authors:** Effie Chew, Wei-Peng Teo, Ning Tang, Kai Keng Ang, Yee Sien Ng, Juan Helen Zhou, Irvin Teh, Kok Soon Phua, Ling Zhao, Cuntai Guan

**Affiliations:** ^1^Division of Neurology, Department of Medicine, National University Hospital, Singapore, Singapore; ^2^Yong Loo Lin School of Medicine, National University of Singapore, Singapore, Singapore; ^3^National Institute of Education, Nanyang Technological University, Singapore, Singapore; ^4^School of Exercise and Nutrition Sciences, Institute for Physical Activity and Nutrition, Deakin University, Melbourne, VIC, Australia; ^5^Institute for Infocomm Research (I^2^R), A*STAR, Singapore, Singapore; ^6^Department of Rehabilitation Medicine, Singapore General Hospital, Singapore, Singapore; ^7^Center for Sleep and Cognition, Center for Translational MR Research, Yong Loo Lin School of Medicine, Singapore, Singapore; ^8^Neuroscience and Behavioral Disorders Program, Duke-NUS Medical School, Singapore, Singapore; ^9^School of Medicine, University of Leeds, Leeds, United Kingdom; ^10^School of Computer Science and Engineering, Nanyang Technological University, Singapore, Singapore

**Keywords:** stroke, motor recovery, transcranial direct current stimulation, brain-computer interface, motor imagery

In the published article, author Kok Soon Phua had a wrong affiliation. Instead of affiliation 4, they should have affiliation 5.

The authors apologize for this error and state that this does not change the scientific conclusions of the article in any way. The original article has been updated.

